# Impact of Manual Addition of Vancomycin to Polymethylmethacrylate (PMMA) Cements

**DOI:** 10.3390/antibiotics13080721

**Published:** 2024-08-01

**Authors:** Clemens Kittinger, Michael Eder-Halbedl, Klaus Dieter Kühn

**Affiliations:** 1 Development and Research Insitute of Hygiene, Microbiology and Environmental Medicine, Hygiene Medical University of Graz, Neue Stiftingtalstraße 6/III 1, 8010 Graz, Austria; clemens.kittinger@medunigraz.at; 2 Department of Orthopedics and Trauma, LKH-Feldbach, Ottokar-Kernstock-Straße 18, 8330 Feldbach, Austria; michael.eder.halbedl@gmail.com; 3 Department of Orthopaedics and Trauma, Medical University of Graz, 8010 Graz, Austria

**Keywords:** PMMA, manually added vancomycin, mechanical properties, efficacy, elution, Copal G+V, VancogenX

## Abstract

(1) Background: The addition of antibiotics to bone cements is a common practice in the treatment of periprosthetic joint infections. In revision cases, the amount and type of antibiotic is often insufficient and additional antibiotics must be added. The addition, however, changes the product itself, and the surgeon becomes the “manufacturer” of the bone cement. PMMAe wished to clarify whether the admixture of antibiotics changes the mechanical stability of the bone cements used and if the added antibiotics were still functional and released in sufficient quantities. (2) Methods: We compared two industrially manufactured vancomycin-containing PMMA cements; the low-viscous VancogenX^®^ (TECRES, Sommacampagna, Italy) and the high-viscous Copal^®^ G+V (Heraeus Medical GmbH, Wehrheim, Germany), with two PMMA cements loaded with aminoglycosides, to which 2.0 g of vancomycin (Hexal CT1631) were manually added—the high-viscous Smartset^®^ GHV and the medium-viscous Antibiotic Simplex with Tobramycin (antibiotic Simplex^®^ T). Test specimens of the bone cements were used to determine mechanical stability (bending strength and bending module), and the release of the antibiotics was determined by HLPC and modified Kirby–Bauer assays. (3) Results: All tested bone cements showed an initial high release within the first hours. Repeated testing after 24 h showed a reduced efficacy of VancogenX^®^ and Smartset^®^ GHV in Kirby–Bauer assays. Long-time release over days showed a release of functional antimicrobial active ingredients over this period of time in anti-microbial assays, but no activity of VancogenX^®^ from day 21 onward. No significant differences in the ISO bending modules could be detected, but in contrast to the bending module, the ISO bending strength was substantially reduced by 10–15 mPal in comparison to both cements of the reference group. The Simplex^®^T met just the ISO 5833; the Smartset^®^ GHV did not after adding vancomycin. (4) Conclusions: In conclusion, the manual addition of 2 g of vancomycin to 40 g of PMMA powder is recommended for the treatment of methicillin-resistant staphylococci. Vancomycin is released over a period of 42 days with concentrations above the MIC for typical staphylococci. The mechanical properties of the PMMA just met, or did not fulfill, ISO mechanical specification. Copal^®^ G+V showed a better elution than VancogenX^®^ over time.

## 1. Introduction

Antibiotic-loaded polymethylmethacrylate (PMMA) cements (ALBCs) enable high local drug concentrations and thus facilitate the prevention and eradication of bone infections with minimal systemic side effects [1,2,3]. The local application of antimicrobial substances is a complementary therapeutic measure to the surgical and systemic antimicrobial treatment in the management of periprosthetic joint infections (PJI) [4,5].

The selection of antibiotics added to PMMA cement is based on the susceptibility of isolated micro-organisms [6]. Vancomycin is primarily used in the treatment of methicillin-resistant *Staphylococcus aureus* (MRSA) and coagulase-negative staphylococci, but it is active against several other Gram-positive bacteria, including streptococci and enterococci [7,8,9].

Recommendations regarding admixing antibiotics into PMMA are predominantly based on mechanical and microbiological in vitro testing, but clinical data are largely lacking [10,11,12]. In addition, the customized addition of antibiotics enables the selection of specific antibiotics directed against isolated bacterial species [13]. For revision surgery, vancomycin-containing cements and cements with manually added antibiotics are mainly used [14,15].

The release of manually incorporated individual antibiotics into cement powder is influenced by several factors. The inhomogeneous distribution of the antibiotic particles in the cement results in non-reproducible release kinetics [14,15,16,17,18]. Furthermore, added antimicrobials might interfere with the working properties and the mechanical behavior of the cement [17,19,20,21,22,23]. The manual addition of vancomycin powder to the PMMA powder before mixing the cement dough allows for a homogeneous distribution in the spacer [10,24,25,26,27]. In order to achieve high local antimicrobial concentrations, the so-called superficial vancomycin coating technique (SVC, an application of vancomycin on the surface of the hardening cement) was established [28].

Importantly, besides synergistic properties [26,29,30,31,32,33,34], antagonistic effects have been described [35,36,37] when using antibiotics in combination with vancomycin [38,39]. Ho et al. (1986) and Smith et al. (2004) described the antagonistic activity of vancomycin in combination with clindamycin against MRSA [40,41]. The combination of gentamicin and vancomycin is characterized by high synergism [38,42].

The manual addition of antimicrobial agents to PMMA cements should be restricted to conditions where no commercial antibiotic-containing PMMA cement is available [43]. As a legal consequence of the customized addition of antibiotics, the surgeon becomes the “manufacturer” of the product, changes the original recipe and thus assumes liability for the product quality and possible consequences.

The aim of this in vitro study is to investigate the manual admixture of vancomycin to PMMA cement and its consequences on the cement properties such as the mechanical stability and release of antibiotics in comparison to industrially manufactured vancomycin-containing cements. The results may serve as the basis for future recommendations for the individual addition of vancomycin to PMMA cements.

## 2. Results

**Figure 1:**
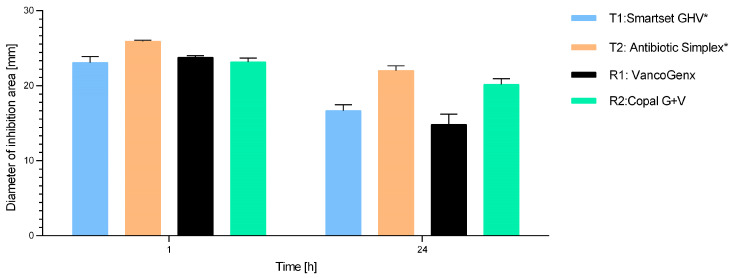
Diameters of inhibition areas of the different cements with G and V addition (* means manually added Vancomycin) tested with *S. aureus* DSM 799 after 1 and 24 h of applying the disks on the agar plates. Values are given as mean with standard deviation.

Agar Diffusion testing for early release (within 24 h). The results of the agar diffusion tests with methicillin-susceptible *S. aureus* (DSM 799) after 1 h and 24 h of elution are shown in Figure 1. The inhibition zones of all tested cements in the modified Kirby–Bauer assay were similar within the first hour, showing no significant difference regarding the early release of antibiotics. After transfer into fresh buffer and 24 h of incubation, all inhibition zones were reduced compared with the samples after 1 h, mainly for VancogenX^®^ and SmartSet^®^ GHV.

**Figure 2:**
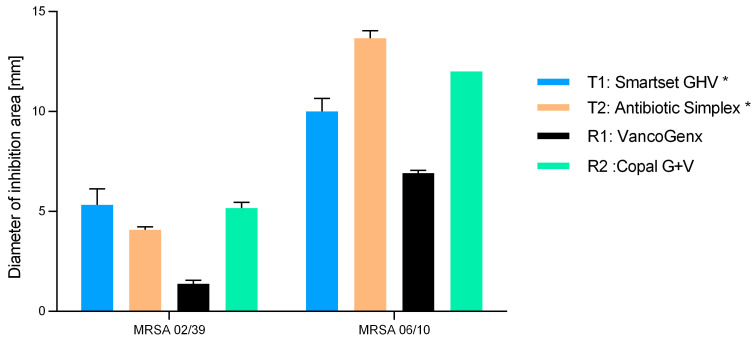
Diameters of zone of inhibition areas of the 24 h eluates of the tested cements against a vancomycin-susceptible MRSA (02/39) and a vancomycin- and gentamicin-susceptible MRSA. Values are given as mean with standard deviation. * means manually added Vancomycin.

Samples taken after 24 h of elution were exposed to different methicillin-resistant *S. aureus* isolates (resistant to G but susceptible to V (MRSA 02/39)) and susceptible to both antibiotics (MRSA 06/10) (Figure 2). All vancomycin-containing cements showed zones of inhibition against the vancomycin-susceptible MRSA strains. When testing with the MRSA 06/10, greater zones of inhibition were documented, reflecting the synergistic effect of gentamicin and vancomycin in case of susceptibility to both substances.

**Figure 3:**
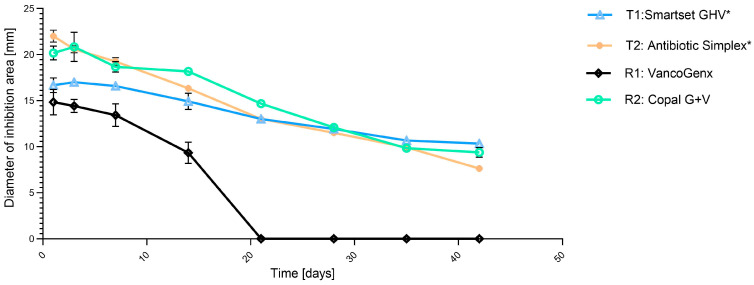
Diameters of inhibition areas of the tested cements from day 1 to day 42 against *S. aureus* DSM 799. Values are given as mean with standard deviation. * means manually added Vancomycin.

Agar diffusion testing for long term release. Zone of inhibition investigations for methicillin-susceptible *S. aureus* over a period of 42 days are shown in Figure 3. VancogenX^®^ (R1) had the lowest zone of inhibition diameters over 42 days, and after day 21, no antimicrobial activity was detectable. Both cements of the test group and Copal^®^ G+V (R2) showed similar zone of inhibition diameters over the whole test period.

**Figure 4:**
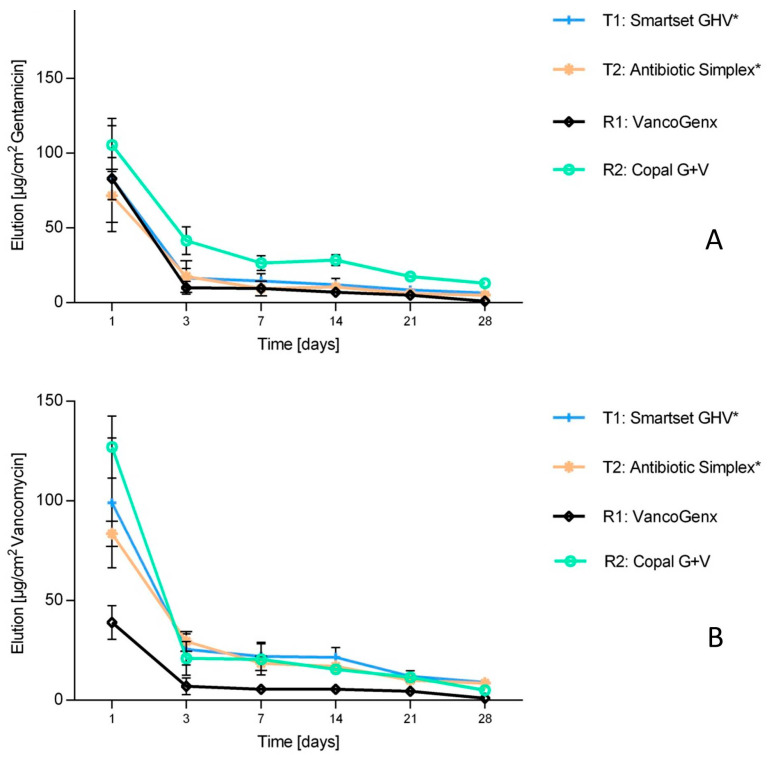
Long-term elution of gentamicin (**A**) and vancomycin (**B**) determined by HPLC. Values are given as mean with standard deviation. * means manually added Vancomycin.

HPLC-detected gentamicin and vancomycin release. All cements had an initial high release of gentamicin after 24 h (Figure 4A). The gentamicin elution of VancogenX^®^ (R1) was initially only high within the first 24 h, but from day three on, the elution was only half or less compared to the other bone cements. This was similar to the microbiological investigations, where areas of inhibition were not detectable from day 21 on for VancogenX^®^ (R1) also (Figure 3). Copal^®^ G+V with the lowest amount of gentamicin showed the best gentamicin release. The release of vancomycin was also lower for VancogenX^®^ (R1) compared to the other bone cements over the tested period of time (Figure 4B). Copal^®^ G+V and manually mixed vancomycin-containing SmartSet^®^ GHV as well as Simplex^®^T showed a comparable elution of vancomycin in this combination.

**Figure 5:**
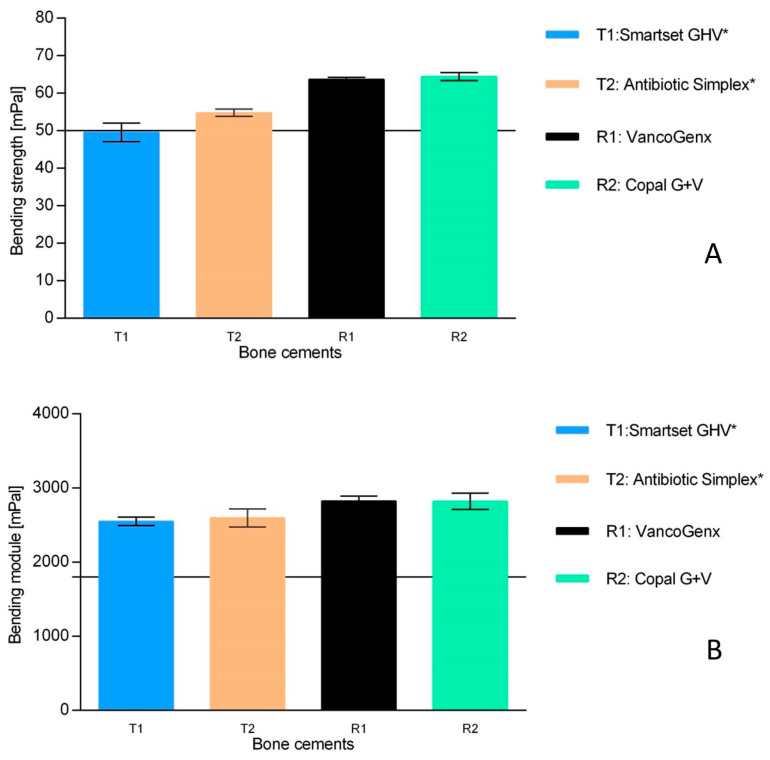
Chart of the ISO bending strength (**A**) and of the bending module (**B**) of the designated cements. The bars represent the bending strength [MPa]. The horizontal line indicates in (**A**) and 1800 MPa in (**B**), which are the limiting values for bending strength and bending modules (bars are given with the mean and standard deviation of the mean, representing six single measurements according to ISO 5833). * means manually added Vancomycin.

Mechanical properties. The ISO bending strength of Antibiotic Simplex^®^T (T2) marginally met the specification, and Smartset^®^ GHV+2gV (T1) did not fulfill (Figure 5A).

No significant differences in the ISO bending modules could be detected, but the ISO bending strength was substantially reduced by 10–15 MPa in comparison to both cements of the reference group (Figure 5B).

## 3. Discussions

The type of the admixed antibiotic has a considerable impact on physical and mechanical properties of the used cement [1,12,13,14,15,44]. Vancomycin is specifically added to PMMA cement in the treatment of oxacillin/methicillin-resistant staphylococci in revision surgeries. Whenever vancomycin is individually hand-mixed into the cement, the maintenance of required properties such as homogeneity, mechanical stability, safety, release profile, and efficacy is of paramount relevance. The combination of vancomycin and gentamicin is widely used as it covers a broad antimicrobial spectrum, including MRSA, and its synergistic behavior was shown to be beneficial [45]. In vitro data suggest that cement spacers loaded with aminoglycoside and glycopeptide antibiotics inhibit susceptible bacteria for 4–6 weeks [8,9,46]. Paz Jiménez et al. (2015) tested the elution of cefazolin and vancomycin and confirmed a much higher elution of cefazolin than vancomycin but a lower strength compared to manually added vancomycin [12].

If lyophilized clumpy vancomycin powder is added to PMMA powder, the pulverization of the antibiotic with a mortar to obtain a homogeneous mixture under “sterile” conditions is required [45,47,48]. Therefore, it is helpful to use a standard operating procedure for mixing in order to reduce all factors which can influence the homogeneity and quality of the cement properties [49,50].

In the present study, adding 2 g of vancomycin showed no significant difference in the early efficacy until 24 h. Gallo et al. (2013) found a superiority in vancomycin release with low-viscous VancogenX^®^ (R1) compared to three high-viscous brands after manually adding vancomycin within hours to a few days. Vancomycin is a slow bactericidal antibiotic which eradicates bacteria much slower than other anti-infective agents (Small and Chambers 1990). In this study, VancogenX^®^ (R1) showed a distinct lower efficacy over time. VancogenX^®^ (R1) had the lowest efficacy of all cements over 42 days, and between day 14 and day 21, the zone of inhibition vanished completely. This may be explained by the fact that VancogenX^®^ (R1) contains only 1 g of vancomycin (compared to 2 g of the other tested cements) and the cement matrix has more hydrophobic polymers than Copal G+V (R2) and Smartset GHV (T1). This might result in a lower absorption of liquids which leads to a reduced elution of antibiotics during the diffusion process. In addition, the quality of the incorporated antibiotics might influence the elution as well as the efficacy of the released substance. Antibiotic Simplex^®^ (T2) with 2.5% tobramycin and 2 g of manually added vancomycin also contains hydrophobic polymers but the release efficacy is significantly higher than VancogenX^®^ (R1). Antibiotic Simplex T+2gV (T2), Smartset^®^ GHV+2gV (T1) and Copal^®^ G+V (R2) showed a continuous constant release as well as efficacy over the whole tested period. The higher efficacy of commercially available cements compared with manually mixed antibiotics to cements was previously described by Ferraris et al. (2010) [16].

Smartset^®^ GHV + 2 g V (T1) and Copal^®^ G+V (R2) behaved similarly during the whole test period although the cements contained different concentrations of gentamicin (1 g in Smartset^®^ GHV (T1) versus 0.5 g in Copal^®^ G+V (R2)). The highly comparable behavior of these cements might be a result of a successful manual admixture of vancomycin to Smartset^®^ GHV (T1).

Neut et al. (2003) compared the industrial addition of gentamicin with manual mixing on three PMMA cements with different viscosities. In all cases, the industrial adding showed a significantly better release of 22% for the low viscous CMW^®^ 3 and 65% for both the high-viscous Palacos^®^ R and the medium-viscous Palamed^®^ (=Palacos^®^ MV). To our knowledge, an inhomogeneous distribution of antibiotics in PMMA cement can lead to a comparatively high elution, but low mechanical properties [18].

All tested vancomycin-containing cements showed zones of inhibitions against all clinically vancomycin-susceptible MRSA strains. The lowest efficacy was observed with VancogenX^®^ (R1). When testing the 24 h eluates against MRSA, all cements showed comparable results in their release with the exception of Smartset^®^ GHV (T1) and VancogenX^®^ (R1). The diameters of the inhibition areas were significant smaller (*p* < 0.001) compared to the other cements, and also, the activity against MRSA was impaired. The lower content of vancomycin cannot only explain lower efficacy, as VancogenX^®^ (R1) contains a higher amount of gentamicin combined with its synergistic effect for efficacy and release.

The synergistic effects of gentamicin and vancomycin usually lead to a better elution of both antibiotics [30]. Gentamicin inhibits the ribosomal functioning, and the target of vancomycin is the bacterial cell wall. Both antibiotics in combination increase antimicrobial activity [1,51,52,53] and increase the local concentration of both antibiotics [42,54].

It was shown that vancomycin shows as low a diffusion as gentamicin out of PMMA because of their physical–chemical properties, their molecular weight, their interaction with body liquids and their temperature sensitivity in combination with cement properties like polymer matrix composition, porosity and roughness, as well as the mixing and adding manner [42,55].

In this study, the long-term efficacy results corresponded well with the elution results of all tested cements. This is comparable with clinical observations after removing Copal^®^ G+C+V spacer six weeks after implantation. Fink et al. (2011) determined the antibiotic concentration of the connective tissue between the spacer and the adjacent bone tissue [56]. Sufficient antibiotic concentrations (5 to more than 100 µg) were found to be higher per gram of tissue than the MIC for gentamicin, clindamycin and vancomycin. These results corresponded well with the in vitro data for Copal^®^ G+C+V of Malhotra et al. (2018) [13].

Anagnostakos et al. (2006) tested the release characteristics of a gentamicin–vancomycin-loaded PMMA spacer (1 g of gentamicin/4 g of vancomycin in 80 g of PMMA) under in vitro and in vivo conditions [57]. There was less elution of antibiotics in vivo than in vitro. Sufficient antibiotic elution and growth inhibition were also observed for approximately 2 weeks on an explanted PMMA spacer. Spacers are only effective under in vivo conditions if the incorporated antibiotics release sufficiently [58]. An instant high release only within the initial phase after implantation is not adequate to eliminate bacteria, and the recolonization of such spacers cannot be excluded [59].

The addition of antibiotics to PMMA cement powder is known to influence the cement’s mechanical properties [13,19,54,60,61,62]. This influence on mechanical properties after adding antibiotics to PMMA varies from drug to drug and depends significantly on the manner of admixing antimicrobial substances to PMMA as well as the cement brand used [63,64,65,66].

As the antibiotic particles are not incorporated in the polymer matrix, they are a foreign body in the cured cement, and this is necessary for their release. Especially for mechanical reasons, it is crucial that the quantity of 4 g of antibiotics per 40 g of cement powder is not exceeded [10]. No significant differences in the ISO bending modules could be detected in the study, but the ISO bending strength of the test group was substantially reduced by approximately 20% in comparison to the references not altered [42]. A high reduction in the ISO bending strength of Smartset GHV (T1) from approximately 66 MPa to 49.4 MPa after the manual addition of 2 g V was reported by [19].

In contrast to the addition of antibiotic powder, Hsieh et al. (2009) successfully added liquid gentamicin to a vancomycin-containing cement dough and observed a synergistic effect on the release properties of the antibiotics [32,67,68]. The addition of 2 g of antibiotics to 40 g of cement powder generally offers a satisfactory release of vancomycin and acceptable compression mechanical properties. The ISO compression and ISO bending modules are often not reduced after adding antibiotics [62]. Rosslenbroich et al. (2012) tested the influence of the manual adding of daptomycin to PMMA [69]. There was an increase in the bending modules, and a low influence on bending strength only when 7.5% daptomycin was added, but also no effect in compression. Similar results were found by Humez et al. (2023) [65]. Gölge et al. (2014) found that the addition of teicoplanin and ciprofloxacin adversely affected mechanical properties [60].

## 4. Materials and Methods

PMMA cements. We compared two industrially manufactured vancomycin-containing PMMA cements (reference group, low-viscous VancogenX^®^ and high-viscous Copal^®^ G+V; see Table 1) with two PMMA cements loaded with aminoglycosides, to which 2.0 g of vancomycin (Hexal CT1631) was manually added (test group, high-viscous Smartset^®^ GHV and medium-viscous Antibiotic Simplex with Tobramycin (antibiotic Simplex^®^ T)).

PMMA cement test specimens. Small rectangular pieces of bone cement, 15 × 10 × 3.3 mm (Dynstat™ specimens, Zwick, Ulm, Germany), according to DIN standard 53435 for impact testing, were prepared under atmospheric pressure and at a room temperature of (23 °C) with humidity of 34% according to the manufacturers’ manuals. The doughy cement was then immediately transferred to steel molds, covered with a plastic film and hardened according to ISO standard 5833 under a pressure of 5 N. After 20 min, the hardened cement was taken out of the molds and stored in small plastic containers at room temperature until use (24–48 h). The mean weight (Cubis Sartorius, Wien, Austria) of the specimen was 0.6104 g with a standard deviation of 0.0186 g (3.04%).

Elution of the PMMA cements for microbiological and high-performance liquid chromatography (HPLC) investigations. The release of antibiotics from PMMA cements was measured using agar diffusion tests and HPLC [70] after different periods of elution. Dynstat™ specimens were eluted in 10 mL of phosphate-buffered saline (PBS, Biochrom, Berlin, Germany). The test specimens were dropped in, swayed once and incubated at RT. At the desired time points, the specimens were removed and transferred into a new vial with fresh buffer. Elution was carried out over a period of 28 days (HPLC) and 42 days (microbiological tests), and specimens were transferred after day 1, 3, 7, 14, 21, 28, 35 and 42. Buffers with eluted antibiotics were kept at 4 °C until measurement (24 h). Experiments were carried out in triplicates.

Test strains. Clinical isolates were used, two methicillin-resistant *S. aureus* (MRSA 02/39 and MRSA 06/10) and one methicillin-susceptible *S. aureus* (DSM 799). The *S. aureus* strains used for agar diffusion testing and their minimal inhibitory concentrations (MIC) for gentamicin and vancomycin are shown in Table 2.

Bacteria at 0.5 McFarland were plated onto Mueller Hinton agar plates (according to EUCAST, Becton Dickinson, Vienna, Austria). After plating the bacteria suspension, a 6 mm diameter hole was pricked into the middle of the plate reaching the bottom of the plate. A total of 50 µL of the eluates was transferred into the hole for the agar diffusion test. The agar plates were then incubated at 37 ± 1 °C for 24 h. The areas of inhibition were always measured directly after incubation. The diameters were measured twice (second measurement perpendicular to the first), and the mean was calculated. Eluates of three different specimens were tested. Long-term release was tested against MSSA to achieve the highest sensitivity and detectability in case of low antibiotic concentration in the eluate.

Determination of gentamicin and vancomycin concentration in elution samples. The determination of the gentamicin and vancomycin concentration was performed on a modular HPLC 1200 Series (Agilent Technologies, Waldbronn, Germany) using a Luna C18 (II) column, 150 × 2 mm, with two C18, 4 × 2 mm, guard columns (Phenomenex, Aschaffenburg, Germany) thermostated at 25 °C (gentamcin) and 40 °C (vancomycin), respectively. The HPLC method used was previously described by Heller et al. [70]. The chromatograms were evaluated with an Analyst (Applied Biosystems, Darmstadt, Germany), and concentrations were calculated with Excel (Microsoft, Unterschleißheim, Germany).

Determination of tobramycin concentration in elution samples. Chromatographic separation was performed on a modular HPLC 1200 Series (Agilent Technologies, Waldbronn, Germany) using a Luna C18 (II) column, 150 × 2 mm, with two C18, 4 × 2 mm, guard columns (Phenomenex, Aschaffenburg, Germany) thermostated at 25 °C. The detection of tobramycin and internal standard was carried out using an API 4000 QTrap (Applied Biosystems, Darmstadt, Germany). Ionization was carried out with an electrospray interface (positive polarity) using a mass selective detector in multiple reaction monitoring mode (MRM). The chromatograms were evaluated by the software Analyst 1.4.2 (Applied Biosystems, Darmstadt, Germany) and calculated with Excel (Microsoft, Unterschleißheim, Germany). Standard solutions of the respective antibiotics were used as calibration standards. All results were calculated in µg/cm², and values are expressed as mean with the standard deviation of the mean.

Determination of the mechanical properties according to ISO standard 5833. To prove both the stability and bending ability of the tested bone cements, the mechanical properties were tested according to ISO standard 5833 “Implants for surgery—Acrylic resin cements” in a four-point bending test, performed with a universal testing machine (Zwick Roell, Ulm, Germany). Thus, the test specimens were forced until breakage (F_max_, maximum force) or investigated for their ability to endure a test force with minimal deformation. Rectangular specimens (3.3 × 75.0 × 10.0 mm) were loaded with a constant crosshead speed of 5 mm/min. The tests were run at 23 ± 1 °C with dry specimens prepared 24 h before testing. The four-point test fixative had a distance of 60 mm between the outer loading points and a distance of 20 mm between the inner loading points. The tests were continued until failure, and the maximum force was used to calculate the bending strength. The bending module was calculated from the difference between the deflections under the loads of 15 N and 50 N. All measurements were carried out three times with different specimens.

Statistics. Microbiolocical data presentation, statistics and graphs were carried out with GraphPadPrism™ 5.01 for Windows, GraphPad Software, San Diego, CA, USA, www.graphpad.com. A two-sided *p*-value of <0.05 was considered significant. Statistical data for mechanical testing followed ISO 5833.

## 5. Conclusions

In conclusion: the manual addition of 2 g of vancomycin to 40 g of PMMA powder is recommended for the treatment of methicillin-resistant staphylococci. Vancomycin releases over a period of 42 days with concentrations above the MIC for typical staphylococci without compromising the mechanical properties of the PMMA and therewith meets the specification of ISO/ASTM standards. Copal G+V showed significant better elution than VancogenX over time.

## Figures and Tables

**Table 1:** Tested PMMA cements with antibiotic concentration before and after manual addition of vancomycin (G = gentamicin, V = vancomycin, T = tobramycin, HV = high viscous).

Cement Tested	Industrial Added Antibiotics (Per 40 g PMMA)	Additionally (Manually) Added Vancomycin
Test group		
T1: Smartset^®^ GHV	1.0 g Gentamicin	+2.0 g Vancomycin Hexal (CT1631)
T2: Antibiotic Simplex^®^ T	1.0 g Tobramycin	+2.0 g vancomycin Hexal (CT1631)
Reference group		
R1: VancogenX^®^	1.0 g Gentamicin plus 1.0 g Vancomycin	no
R2: Copal^®^ G+V	0.5 g Gentamicin plus 2.0 g Vancomycin	no

**Table 2:** The minimal inhibitory concentrations (MIC) of the tested staphylococcal strains for gentamicin, tobramycin and vancomycin.

Antibiotic	Methicillin-Resistant *S. aureus* (MRSA 02/39)	Methicillin-Resistant *S. Aureus* (MRSA 06/10)	Methicillin-Susceptible *S. aureus* (DSM 799)
Gentamicin	>256 µg/mL (R)	0.5 µg/mL (S)	0.5 µg/mL (S)
Vancomycin	0.75 µg/mL (S)	1 µg/mL (S)	0.5 µg/mL (S)
Tobramycin	>256 µg/mL (R)	0.5 µg/mL (R)	0.5 µg/mL (S)

Note. S (susceptible) and R (resistant) in brackets refer to the interpretation of the MIC according to EUCAST. Susceptibility was determined by using the Epsilometer test (E-test, Becton Dickinson) according to the manufacturer’s guidelines.

## Data Availability

All data are presented in this article. The data are also available from the Medical University of Graz (MUG).
